# Practice Site Heterogeneity within and between Medicaid Accountable Care Organizations

**DOI:** 10.3390/healthcare12020266

**Published:** 2024-01-20

**Authors:** Zachary Dyer, Matthew Alcusky, Jay Himmelstein, Arlene Ash, Michaela Kerrissey

**Affiliations:** 1 Department of Population and Quantitative Health Sciences, UMass Chan Medical School, Worcester, MA 01655, USA; 2 Department of Health Policy and Management, Harvard T.H. Chan School of Public Health, Boston, MA 02115, USA

**Keywords:** accountable care organizations, Medicaid, Massachusetts, healthcare reform, cluster analysis

## Abstract

The existing literature has considered accountable care organizations (ACOs) as whole entities, neglecting potentially important variations in the characteristics and experiences of the individual practice sites that comprise them. In this observational cross-sectional study, our aim is to characterize the experience, capacity, and process heterogeneity at the practice site level within and between Medicaid ACOs, drawing on the Massachusetts Medicaid and Children’s Health Insurance Program (MassHealth), which launched an ACO reform effort in 2018. We used a 2019 survey of a representative sample of administrators from practice sites participating in Medicaid ACOs in Massachusetts (*n* = 225). We quantified the clustering of responses by practice site within all 17 Medicaid ACOs in Massachusetts for measures of process change, previous experience with alternative payment models, and changes in the practices’ ability to deliver high-quality care. Using multilevel logistic models, we calculated median odds ratios (MORs) and intraclass correlation coefficients (ICCs) to quantify the variation within and between ACOs for each measure. We found greater heterogeneity within the ACOs than between them for all measures, regardless of practice site and ACO characteristics (all ICCs ≤ 0.26). Our research indicates diverse experience with, and capacity for, implementing ACO initiatives across practice sites in Medicaid ACOs. Future research and program design should account for characteristics of practice sites within ACOs.

## 1. Introduction

Accountable care organizations (ACOs) have emerged as a primary mechanism for shifting incentives from fee-for-volume towards fee-for-value by promoting integrated care and holding groups of providers responsible for the cost and quality of care for a defined population. Spurred by policies such as the 2010 Patient Protection and Affordable Care Act (ACA) and the 2015 Medicare Access and Children’s Health Insurance Program (CHIP) Reauthorization Act (MACRA), over 1000 ACOs have been established since 2010. ACO contracts cover more than 40 million lives today [1,2,3]. Amid this overall growth, ACOs have arisen in Medicaid programs, which face unique opportunities and challenges in integrating care and holding providers accountable for managing populations with high levels of clinical and social complexity [4].

Medicaid, insuring one-quarter of all Americans, is a critical safety net for low-income Americans and an important lever in large-scale efforts to improve healthcare and public health in the US. Policy innovations, such as value-based care models, Medicaid managed care programs, and accountable care organizations, have been introduced to many state Medicaid programs to improve the efficiency and quality of healthcare delivery, representing a concerted effort to adapt and improve Medicaid’s ability to serve the evolving needs of diverse populations, and serving as a catalyst for broader change within the healthcare system. To date, more than a dozen state Medicaid programs have adopted an ACO strategy, and even more seek to incorporate ACOs in the future [4,5,6].

Evidence of ACOs’ ability to generate high-quality care while curbing costs is growing but remains limited [7,8,9,10,11,12]. Evaluations and academic studies have shown modest cost savings of up to USD 500 per beneficiary per year after several years in an ACO [8,11,13,14]. A systematic review of Medicare, Medicaid, and commercial ACOs reported consistent associations between ACOs and reduced inpatient utilization, fewer emergency visits, and improved quality of care [15]. Early appraisal of the performance of Medicaid ACOs in four states showed significant reductions in emergency department visits for some ACOs, but little impact on health expenditures [16]. Some ACOs achieve considerable success, while others withdraw from ACO programs entirely, which can be due to features (or flaws) in program design driving selection into and out of ACO programs [3,14,17].

Amid the growing literature on ACO program outcomes, studies examining the determinants of success and variation in performance remain comparatively sparse. Taxonomies characterizing ACOs by leadership (physician-led, hospital-led, etc.), experience with value-based payments, population management strategies, or risk-sharing (upside risk, downside risk, etc.) exist [18,19,20,21], but these do not consistently predict ACOs’ effectiveness [19,20,22]. Some studies have found that physician-led ACOs, on average, exhibit greater per member savings than hospital-led ACOs [9,23]. However, others have refuted this claim [24] and found more variation within ACO types than between them [19]. In looking for the keys to ACOs’ success, overall organizational characteristics at the ACO level have yet to offer consistent lessons. The implications for health system leaders and policymakers remain unclear.

This paper addresses two shortcomings in the current literature: the lack of studies examining practice-site-level characteristics within ACOs, and the limited number of studies focused on Medicaid program ACOs.

First, one potential issue underlying the varied evidence at the ACO level is the presence of heterogeneity among the practice sites that comprise ACOs. Efforts to characterize ACOs have largely neglected the variety and breadth of experience across practice sites within ACOs. Various incentive structures for care transformation and alternative payment models have been available to practices for more than a decade, from patient-centered medical homes (PCMHs) to commercial and Medicare ACOs [2,25]. Practice sites caring for patients as part of new Medicaid ACOs have varying levels of experience with risk-bearing contracts, different infrastructure for health information technology and population health management, a range of relationships with community-based organizations, and distinct patient populations [15,19]. The extent to which individual practice site characteristics and experiences help explain the variations in program success within and between ACOs has remained unclear.

Second, much of the current ACO literature focuses on commercial and Medicare contracts, which were generally implemented earlier than for Medicaid programs and populations [8,15,18]. Alternative payment models for Medicaid programs face different challenges given their more complex and diverse populations, with a greater burden of behavioral health conditions and health-related social needs [26].

Massachusetts offers an instructive setting for exploring practice site heterogeneity among Medicaid ACOs because of the state’s substantial reform efforts, from which extensive early evidence can be derived. Following a pioneering approach to healthcare cost containment in 2012, Massachusetts’ practices have experienced significant healthcare transformation efforts, including some of the earliest global payment models [27,28,29,30]. More recently, the Massachusetts Medicaid program (MassHealth) contracted with 17 ACOs as a centerpiece of its five-year (2017–2022) Delivery System Reform Incentive Payment (DSRIP) program, seeking to integrate care, introduce cost and quality accountability for providers, and address health-related social needs [31]. Massachusetts now has one of the most extensive Medicaid ACO programs in the country, with nearly all major health systems participating, building on an already robust and inclusive Medicaid program.

In this study, we characterize the variability across practice sites within and between newly established Medicaid ACOs, examine the extent to which practice site characteristics are associated with early progress in the ACO program, and identify ACO-level characteristics that help explain the variability between ACOs. Our goal with this study is to describe heterogeneity within and between ACOs in order to better understand this important policy tool.

## 2. Materials and Methods

### 2.1. Data Collection

For this observational cross-sectional study, we analyzed data from a novel electronic survey of practice sites participating in Massachusetts Medicaid ACOs. The survey was developed by experts within the MassHealth ACO program and at the Public Consulting Group, who were contracted to implement the survey. When relevant, previously validated survey scales were used as part of the overall survey, and cognitive testing was completed prior to the survey’s administration. The survey sought primary care practice site administrators’ (i.e., practice site managers’) perspectives about the first year of ACO implementation and documented site-level organizational characteristics. It collected information about experiences with alternative payment models, understanding of and performance in the MassHealth ACO program, specific care delivery strategies used, the degree of standardization within the practice, satisfaction with the program, and other practice site characteristics. All measures were self-reported. The respondents were informed that these early-stage data would not be used to evaluate individual site performance or affect financial disbursements in any way. Access to these data for academic publishing was provided by MassHealth and the Public Consulting Group. This study is part of a larger project, the independent evaluation of the Massachusetts Medicaid DSRIP program [31], which was classified as “not human subjects research” by the UMass Chan Medical School’s IRB.

The representative sample was drawn from a list of all practice sites participating in one of 17 Massachusetts Medicaid ACOs as of August 2018 (*n* = 929). Of these, 248 were excluded from sampling due to data limitations or lack of relevance, as well as 77 sites with unknown numbers of assigned members, 66 with fewer than 50 members, 52 that joined the ACO program after the DSRIP program started (March 2018), 38 single-physician sites, 10 acute-care-only sites, and 5 sites outside of Massachusetts in bordering areas. Sites with fewer than 50 members accounted for only 1.2% of MassHealth members, limiting their exposure to the program. These criteria led to a sampling frame of 681 sites, from which research sites were drawn randomly, stratified by ACO; up to 30 were drawn per ACO, and after further excluding 9 sites that had merged or were no longer part of the program when contacted, there was a final sample of 353 unique practice sites. These 353 sampled practice sites had over 500,000 attributed members—well over half of the nearly 900,000 members participating in the program at the time.

A practice site administrator completed the survey for each group practice or health center (i.e., “practice site”). The practice site administrators’ contact information was collected from ACO representatives. The survey was administered in July through September 2019, with a response rate of 64% (225 out of 353 sites surveyed). Within the 17 ACOs under which the sites are organized, the practice site response rates ranged from 42% to 100%. The survey was timed to reach the practice sites 16-20 months after the program’s implementation began in March 2018.

Data on practice site type (i.e., group practice or health center) and the number of attributed members were obtained from MassHealth; the classification of ACO anchoring organizations is described in organizational profiles published by the Massachusetts Health Policy Commission in April 2019 [32]. ACO size was categorized into tertiles of attributed MassHealth members (small: <19,000 members; medium: 19,000–29,000 members; large: >29,000 members).

### 2.2. Contract Participation Measures

To characterize prior experience with cost accountability contracts, we analyzed three survey-reported measures: (1) whether the practice had about half or more of patients covered under any contracts with cost-of-care accountability; (2) whether the practice had any past participation in an ACO contract, including Medicare upside-only risk-bearing contracts, Medicare two-sided risk-bearing contracts, or commercial ACO contracts; and (3) whether the practice had participated in another payment contract with the other clinical sites in their current Medicaid ACO. We included these measures to assess the heterogeneity of prior ACO participation within ACOs and to describe the extent to which different levels of prior ACO participation contribute to between-ACO variance.

### 2.3. Process Measures

We used four process variables to measure practice site progress in the ACO program: (1) “process change”, an overall assessment of the extent of change; (2) “standardized practices”, changes in the degree of standardization in care processes and team structure; (3) “ability to care for vulnerable populations”, changes in the difficulty of tailoring the delivery of care to meet the needs of vulnerable populations; and (4) “social service referrals”, changes in the frequency of referrals to social service organizations to meet patients’ needs. Each used a scale of one to five, anchored with descriptions of change, standardization, difficulty, and frequency, respectively, within the prior year. The first two measures address the level of participation in ACO activities, while the second two address progress toward ACO goals.

### 2.4. Outcome Measure

The survey measured sites’ understanding of, attitudes toward, and progress in achieving ACO goals. For this study, we focused on one question: self-reported change in the ability to deliver high-quality care within the prior year, as evidence of perceived program progress at this early stage of implementation. This was measured on a five-point scale from “gotten a lot harder” to “gotten a lot easier”.

### 2.5. Practice Site Characteristics

Practice site characteristics included practice site type (characterized as either health center or group practice), whether the practice serves adult, pediatric, or both patient populations, and whether the practice serves fewer than 500 patients, which is close to the median number of Medicaid patients per site.

### 2.6. ACO Characteristics

We included two characteristics of ACOs as covariates: (1) whether it is anchored by a teaching/academic hospital, a community hospital, or a physician group; and (2) the size of the ACO: small, medium, or large.

### 2.7. Analyses

We described all practice sites in the sampling frame and the 225 sites that responded to the survey, using the practice site and ACO characteristics described above. We calculated the frequencies and proportions of responses for the four process measures and one outcome measure. To visualize the variability of the responses across ACOs, we used heatmaps depicting the distribution of practice site responses by ACO for selected measures.

We used multilevel logistic models with ACOs treated as random effects to compare within-ACO heterogeneity versus between-ACO heterogeneity. We first used a model with no predictors to calculate ACO-level variance, intraclass correlation coefficients (ICCs), and median odds ratios (MORs) for each practice site’s contract participation, process, and outcome measure. The ICC measures the proportion of the variance in practice site responses that can be attributed to differences at the ACO level [33,34]. The MOR measures the heterogeneity in practice site responses between ACOs [33,34]. The MOR represents the median increased odds of a practice site response being lower if it were in a different ACO.

We define heterogeneity in this study as the degree of variability in practice site characteristics, progress toward program goals, and the achievement of program outcomes. “Within-ACO” heterogeneity is how different practice sites within an ACO are from one another. “Between-ACO” heterogeneity is how different the practice sites in one ACO are from practice sites in another ACO. If all practice sites in an ACO had the same characteristics and all practice sites in a different ACO had the same characteristics, but different than those in the first ACO, then they would be said to have low within-ACO heterogeneity, but high between-ACO heterogeneity. Likewise, if all of the practice sites within an ACO had a uniform distribution of characteristics and all of the practice sites in a different ACO had the same uniform distribution of characteristics, they would be said to have high within-ACO heterogeneity, but low between-ACO heterogeneity.

We used a random-intercept multilevel ordinal model using practice-site-level variables to predict the cumulative odds of responding lower on the scale of self-reported ability to deliver high-quality care. We included the following variables: practice site type, past ACO contract participation, the proportion of patients covered by risk contracts, adult or pediatric patient population, and the number of MassHealth members, and we included ACO as a random effect. We calculated adjusted variance, ICCs, and MORs from this model and odds ratios for each covariate.

Finally, we built models including both practice-site-level and ACO-level variables for the same outcomes, adding ACO type and ACO size. We again calculated adjusted variance, ICCs, and MORs, as well as odds ratios for all covariates.

We included the process and outcome measures as ordinal variables in our analyses; contract participation measures were binary. For all of the calculated MORs, we used bootstrapping to estimate 95% credible intervals (CrIs). We resampled at a rate of 1 with replacement, maintaining *n* = 225, in 5000 iterations. We then applied our models to each of the 5000 constructed samples and, using the calculated variances, identified the 2.5th and 97.5th percentiles of variance and used them as bounds for the MOR credible intervals.

The data analysis for this paper was generated using SAS software, Version 9.4 of the SAS System for Windows.

## 3. Results

The sample frame sites (*n* = 672), sampled sites (*n* = 353), and responding practice sites (n = 225) had similar distributions of practice type, practice size, and patient population. Respondent and sampled sites similarly demonstrated similar distribution by type of ACO. Among the responding practice sites, there were many variations. Nearly 40% of respondents reported prior contract experience with clinical partners currently in their ACO. Practices also varied widely by the number of attributed MassHealth members, from fewer than 100 MassHealth members to more than 10,000 members (Table 1).

Table 2 shows the distribution of mean responses within ACOs for the outcome measure and the four process measures pertaining to aspects of progress for practice sites operating within the ACOs. The distribution of mean responses shows the variability in the average responses between ACOs and a spread of progress toward achieving the ACO program goals. The measure where the median ACO had the highest practice site mean was standardized care practices (4.18); the measure where the median ACO had the lowest practice site mean was process change in the past year (2.80).

**Figure 1:**
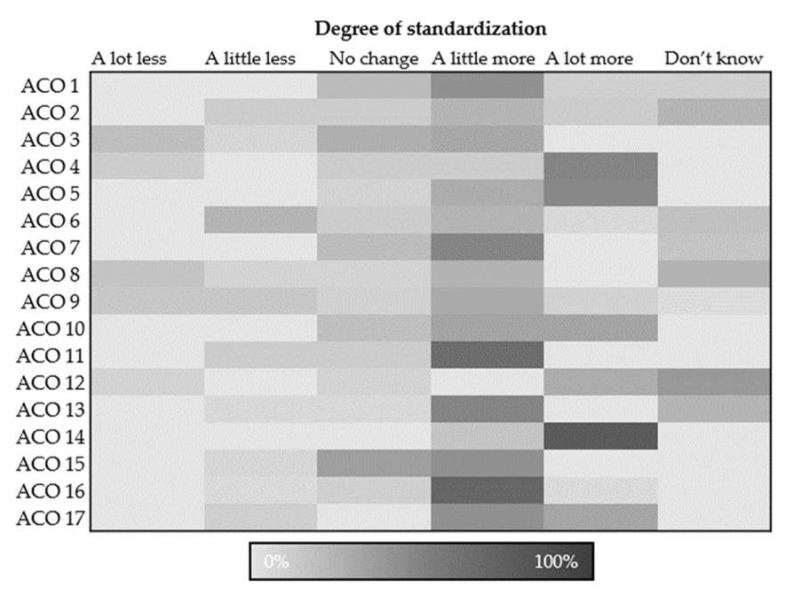
Distribution of responses for perceived standardization of care processes by ACO.

Summary analyses showed substantial variation in responses to most measures within and between ACOs. Figure 1 visually represents the distribution of responses by ACO for one measure, selected for illustrative purposes: care process standardization. Darker shading shows a higher proportion of practice sites that chose that response within their ACO. While some ACOs had a narrow distribution of responses, such as ACO 14, others had a broad distribution of responses, such as ACO 9. Two ACOs’ most frequent response was “no change”, while more (6 of 17) were “a little more standardized” and many (9 of 17) were “a lot more standardized”. This degree of variation in responses by ACO was repeated across most measures.

Table 3 presents the within- and between-ACO variation for contract participation, process, and outcome measures. Contract participation measures varied both within and between ACOs. Previous experience with ACO contracts of any kind showed the highest ACO-level variance (1.13), ICC (0.26), and MOR (2.76 (95% CrI: 2.42–6.53)), meaning that the responses were clustered by ACO for this measure more than for the other analyzed measures.

Two other contract participation measures—past payment contract experience with the same clinical partners, and the proportion of the practice site patient population covered by a contract with cost accountability—showed less correlation by ACO, with ICCs near 0 (0.01 and 0.06, respectively). That is, these measures differed little between ACOs.

ICCs between 0.13 and 0.26 were observed for the four process measures. The MORs for these measures indicate clustering within ACOs, with MORs ranging from 1.94 (95% CrI: 1.64–3.92) for practice standardization to 2.80 (95% CrI: 2.36–5.21) for the ability to care for vulnerable populations. Consistent with the definition of the median odds ratio measure, this association can be interpreted as provided in the following example: an MOR of 2.80 suggests that for a randomly selected practice site the median odds of reporting a better ability to care for vulnerable populations would be nearly three times higher if the practice randomly moved to a different ACO with a better ability to care for vulnerable populations.

Table 4 shows results from the iterative multilevel models that assess the ACO-level clustering of changes in the ability to provide high-quality care after no adjustment (ICC: 0.10), adjustment for practice-site-level measures (ICC: 0.11) and, finally, adjustment for both practice-site- and ACO-level measures (0.01). In the model with practice site characteristics only, several variables appear to be as strong or stronger predictors of changes in the ability to provide high-quality care than ACO, as indicated by odds ratios (ORs) higher than the ACO-level MOR (1.83). Practices that serve both adult and pediatric patients compared to those serving adults alone (OR: 5.11; 95% CI: 2.13–12.23), serving greater than the median number (500) of MassHealth patients per site (OR: 1.77; 95% CI: 0.90–3.47), and practice site type (i.e., group practice versus health center) (OR: 2.23; (95% CI: 0.84–6.48) were similarly or more strongly associated with changes in the reported ability to provide high-quality care than the ACO-level MOR. The variables measuring experience with ACO contracts had weaker associations.

For the model with covariates at both the practice site and ACO levels, the ACO-level MOR was attenuated, with almost all covariates having stronger measures of association than ACO membership, as indicated by odds ratios higher than the MOR of 1.20 (95% CrI: 1.00–4.21). The patient population was most strongly associated with a practice site’s response to their ability to provide high-quality care, with an odds ratio of 5.24 (95% CI: 2.17–12.70) when comparing practice sites that serve both pediatric and adult patients vs. those that serve only adult patients. The anchoring organization was also associated with a practice site’s response. Community-hospital-anchored ACOs had 3.42 times the odds of reporting that it was now easier to provide high-quality care than physician-organization-anchored ACOs.

## 4. Discussion

This study documents substantial heterogeneity of practice site features, experience, and outcomes within Medicaid ACOs, drawing on data from Massachusetts. These characteristics were associated with variations in perceived progress with delivery system reform during the early implementation period of the ACO program. At the same time, our analysis identified practice-level features that exhibit substantial clustering within Medicaid ACOs, indicating that, even while greater heterogeneity exists within Medicaid ACOs than between them, certain practice-level features may meaningfully differentiate ACOs.

Our findings highlight the value of understanding practice-site-level context when studying the use of ACOs as a policy tool. Heterogeneity of performance and organizational characteristics at the ACO level is well documented [7,18,21]. We also found considerable heterogeneity among practice sites within ACOs, particularly with respect to experience with ACO contracts and the process features that we measured.

The differences that we observed among practice sites within ACOs may have important practical implications for policymakers implementing healthcare delivery reform, especially in light of recent guidance from the CMS Innovation Center indicating a long-term commitment to ACOs as a centerpiece of the shift to value-based care and an increased attention to their use in Medicaid programs [35,36]. For example, practices with prior ACO contract experience may be able to build on existing knowledge, provider buy-in, and resources; others could struggle when shifting from a fee-for-service model for the first time but offer great potential for improvement. If so, it may be beneficial in the longer term for payers and policymakers to make it easier for practices to participate (e.g., by limiting risk and providing infrastructure funding) and gain experience as part of any alternative payment model (e.g., Medicaid, Medicare, or commercial). Furthermore, since sites whose all-payer revenue predominantly flows through advanced alternate payment models (APMs) have a larger incentive to redesign their care delivery model to succeed through population health management rather than maximizing fee-for-service volume, policymakers should promote a coordinated multipayer shift towards alternative payment models. Tracking variation in practice-level characteristics can thus help to identify opportunities for improvement and guide decisions about how and where to invest time and resources to better drive outcomes. This requires data at the practice level to understand the context in which reforms are being implemented.

The presence of substantial practice-level variation has practical implications for ACOs and signals a set of choices for ACO leaders and policymakers. For example, some ACOs facing meaningful heterogeneity in their practice sites may choose to invest in infrastructure that will bring all sites to a similar level of readiness for care transformation, such as investments in centralized care management resources housed at the ACO. Others, with a subset of particularly challenged practices, may invest in them disproportionately or establish hybrid models with a mix of centralized and practice-based resources to assist them. Practice-level assessment can also help inform the investments that states make and provide broader evaluations of ACO implementation. For instance, in Medicaid, many practices may be voyaging into APMs for the first time. Others, with experience with ACOs in Medicare or commercial contracts, must identify how to apply other processes to the distinct needs of a Medicaid population and handle competing demands among multiple payers. Indeed, from the practice-level perspective, an ACO is often just one relationship out of many that providers navigate in caring for diverse groups of patients [37].

Our study raises important theoretical implications that require future practice-level research and policy evaluation to identify the features of practices that matter most for successful outcomes in Medicaid ACOs over time. For example, understanding the extent to which practice site homogeneity in processes or other features is an advantage—or not—would be valuable to informing policy. It may be that homogeneity for certain processes is advantageous because it enables the ACO to invest in resources to improve care uniformly. Conversely, heterogeneity for certain processes at the outset may be optimal if it enables the diffusion of knowledge, such that the ACO can help spread learning and facilitate rapid improvement across practices, or tailor experiences to different patient populations. Indeed, there is great variation from state to state in how each Medicaid program is structured, and each state tailors their programming to the needs of their population. This is not dissimilar from federated models of healthcare reform and care integration that are being implemented internationally, such as in Canada, Sweden, and Italy. For example, Italy’s national health service delegates much of the responsibility for implementing healthcare delivery system reform to the regions, and there is substantial variability within and between regions in the approach and pace of restructuring efforts to realize centrally established policy goals, reflecting local needs, attitudes, and resource constraints [38,39,40].

Despite the existence of greater overall heterogeneity within ACOs than between them, practice sites’ responses were meaningfully clustered within ACOs for some measures. For instance, two measures that reflect vital aspects of patient services for Medicaid programs—change in reported ability to care for vulnerable populations, and frequency of referrals to social service agencies—were more strongly differentiated between ACOs than many other measures. This clustering may indicate that some ACOs mobilized change faster than others in these dimensions in the first year of program participation, including through successful partnerships with community-based organizations, as prescribed in the MassHealth ACO program design. It may also reflect that some ACOs brought together practice sites with greater focus and capabilities for serving under-resourced Medicaid populations. This may have important implications for the effectiveness of reform in practice. Future research could deepen our understanding of what transpires in ACO efforts by examining the extent to which certain clustered features within ACOs predict greater success in attaining care quality and cost objectives. This study highlights the gap in our understanding of how practice-level differences correlate with overall readiness for ACO-driven change. Research that fully explores this area could help inform infrastructure investments as a greater number of Medicaid programs integrate ACO models into their approach.

The measure in our study that was least clustered by ACO was the proportion of patients covered by cost accountability contracts. Some practices had all of their patients covered, while others had very few. This is notable for several reasons. For patients, the experience of receiving care from practices on either end of this spectrum may be vastly different even within the same ACO. For clinicians and practice managers, this finding suggests that, within the same ACO, some practices are under greater pressure to achieve performance metrics and better integrate care for their patients, while others may have relatively weak incentives due to low overall ACO enrollment. While the shift to APMs represents an opportunity for Medicaid programs to overcome historical disparities in access, a single effort by Medicaid to drive change in an environment characterized by little other cost accountability may struggle. Inadequate financial incentives for participation in care delivery reform have been cited as a limitation of some ACO programs [41,42].

Indeed, all of the ACOs had a mix of sites with high and low penetration of cost accountability contracts. This highlights a program-wide opportunity for each ACO to prioritize the pursuit of commercial and Medicare ACO contracts for its practices that are still operating with little cost accountability, so as to shift and align incentives. However, increasing a practice’s revenue under cost accountability contracts without multi-stakeholder efforts to align program requirements may overburden sites or force them to pick and choose measures of focus. Additionally, the magnitude and structure of incentives and accountability mechanisms for ACOs and their providers must be carefully considered when evaluating programs’ impact.

In addition to the practice site features described above, the organizational characteristics of size and anchoring institution were associated with self-reported improvements in providing high-quality care during early implementation. Specifically, small ACOs and physician-organization-anchored ACOs were less likely to report improvements in providing high-quality care. Past studies have also found that small Medicare ACOs are more likely to leave the Medicare Shared Savings Program (MSSP) [17,43]. Research studies of the MSSP have established divergent conclusions regarding anchoring institutions. Physician-group-led ACOs were shown to generate greater savings for Medicare [9,44], but also to leave the program at a higher rate [43], raising the question of selection bias [14]. Further research to understand the potentially unique relationships of ACO features and program outcomes in Medicaid ACOs is warranted.

### Limitations

This study has several limitations. First, the data were collected about 18 months into program implementation and only captured early perceived changes, without directly measuring pre- and post-implementation values of measures such as quality of care. Nevertheless, subjective self-evaluation of change in a practice site’s ability to deliver high-quality care is important and relevant. This is especially true for the survey’s respondents, practice site administrators, who tend to be informed of their sites’ cost and quality outcomes. Further research examining specific quality measures will be needed to elucidate which measures may have changed, to what extent, and whether organizations with high (or low) reported changes in quality of care started from higher or lower performance levels. Second, the measures were all from the same survey, making them subject to single-source bias. Third, each state differs in its healthcare and policy landscape, which may limit the generalizability of our findings to other states and other payers. Fourth, we did not have data on a comparison group, and it is possible that the reported changes in care processes were not due to the ACO program and instead reflected secular trends. Fifth, although we had a robust response rate and the characteristics of the respondents resembled those of the overall sample, we do not know whether those who were not sampled and non-respondents differed in their perspectives compared with survey respondents. Finally, our survey was novel and used many different standardized scales. Future research should identify expert-tested scales for measuring practice site characteristics, as well as process and outcome measures, to compare across multiple ACO programs.

## 5. Conclusions

Our findings demonstrate that ACOs and their individual practice sites may start at notably different baselines in population health management infrastructure, resources, incentives, and experiences. This highlights an opportunity for ACOs to pursue targeted payment and delivery system reform strategies customized for the unique features of specific practice sites. Program-wide or ACO-level research may overlook important changes occurring among subgroups of practice sites distinguished by key characteristics. Often, when ACOs are evaluated, they are examined at the individual ACO contract level, without considering the broader context of cost accountability from the practice’s perspective, where much of the desired change must occur. Investments are warranted to increase the availability of practice-level data and to support more comprehensive evaluations that account for heterogeneity in implementation and performance within and between ACOs.

## Figures and Tables

**Table 1:** Practice site characteristics of the survey respondents, sample, and sample frame.

	Respondents (*n* = 225)	Sample (*n* = 353)	Sample Frame (*n* = 672)
Practice type			
Group practice	175 (78%)	278 (79%)	576 (86%)
Health center	50 (22%)	74 (21%)	95 (14%)
Practice size			
<100 members	13 (6%)	28 (8%)	69 (10%)
100–999 members	137 (61%)	214 (61%)	415 (61%)
1000–9999 members	68 (30%)	101 (29%)	176 (26%)
≥10,000 members	6 (3%)	9 (3%)	11 (2%)
Patient population			
Adult patients only	71 (33%)		
Pediatric patients only	41 (19%)		
Both adult and pediatric patients	106 (49%)		
Experience with ACO contracts	55%		
≥50% patients covered by cost accountability contracts	55%		
Contract experience with same clinical partners	39%		
ACO anchoring organization			
Physician-organization-anchored	38 (17%)	66 (19%)	66 (10%)
Community-hospital-anchored	49 (22%)	74 (21%)	75 (11%)
Teaching-hospital-anchored	138 (61%)	213 (60%)	531 (79%)
ACO size			
<19,000 members	62 (28%)	98 (28%)	118 (18%)
19,000–28,999 members	87 (39%)	144 (41%)	397 (59%)
≥29,000 members	76 (34%)	111 (31%)	157 (23%)
ACOs represented	17	17	17
Range of practice sites per ACO	7–25	7–30	7–161
Members attributed to practice sites *(in thousands)*	345	511	824

Notes: Patient population and contract experience were gathered through the survey and, therefore, are only available for survey respondents. Seven (7) practice sites did not respond to the survey item about patient population. Practice type and practice size data were missing for one (1) practice site.

**Table 2:** Survey results: distribution of ACOs’ mean values for process and outcome measures.

1. Process change In the past year, to what extent has your practice changed its processes and approaches to caring for MassHealth members?				
Minimum	Q1	Median	Q3	Maximum
2.00	2.47	2.80	3.19	3.55
2. Standardized practices In the past year, to what degree have care practices and team structure in your clinic become more standardized, less standardized or not changed?				
Minimum	Q1	Median	Q3	Maximum
3.63	3.81	4.18	4.37	4.83
3. Social service referrals How often are MassHealth members referred from your practice to social service organizations to address health-related social needs (e.g., housing, food security)?				
Minimum	Q1	Median	Q3	Maximum
2.80	3.14	3.58	3.91	4.60
4. Ability to care for vulnerable populations In the past year, how has your practice site’s ability to tailor delivery of care to meet the needs of patients affected by health inequities changed?				
Minimum	Q1	Median	Q3	Maximum
2.30	3.00	3.28	3.50	4.14
5. Ability to provide high-quality care In the past year, to what extent has your practice’s ability to deliver high quality care to MassHealth members gotten better, gotten worse, or stayed the same?				
Minimum	Q1	Median	Q3	Maximum
2.63	3.15	3.32	3.44	4.00

Notes: Numbers represent the ranges of ACO-level means. Twenty-three respondents (10%) responded with “Don’t know” to the second listed question. Higher values represent a greater level of change, frequency, or ease. All responses were measured on 5-point scales. Q1 denotes the 25th percentile; Q3 denotes the 75th percentile.

**Table 3:** Measures of ACO clustering and variance.

ACO-Level Variance (SE)		ICC	MOR (95% CrI)	
Contract participation measures				
Experience with ACO contracts	1.13 (0.61)	0.26	2.76 (2.42–6.53)	
Patients covered by cost accountability contracts	0.04 (0.15)	0.01	1.22 (1.11–2.96)	
Contract experience with the same clinical partners	0.22 (0.21)	0.06	1.56 (1.41–3.15)	
Process measures				
Process change	0.63 (0.36)	0.16	2.13 (1.82–4.06)	
Standardized practices	0.48 (0.32)	0.13	1.94 (1.64–3.92)	
Social service referrals	0.71 (0.38)	0.18	2.23 (1.99–4.33)	
Ability to care for vulnerable populations	1.17 (0.55)	0.26	2.80 (2.36–5.21)	
Outcome measure				
Ability to provide high-quality care	0.37 (0.27)	0.10	1.78 (1.57–3.69)	

Notes: ACO = accountable care organization. The ICC (intraclass correlation coefficient) measures the proportion of the variance in all practice site responses that is due to differences at the ACO level. The MOR (median odds ratio) measures heterogeneity in practice site responses between ACOs. Based on the included data, there is a 95% probability that the true MOR lies within the listed credible interval (CrI). Both the MOR and the ICC are derived from the variance, shown here with the standard error (SE), which shows the magnitude of differences in responses across ACOs. Measures of experience are dichotomous variables. Process and outcome measures are on 5-point Likert scales and treated ordinally.

**Table 4:** ACO-level clustering of ability to provide high-quality care after adjusting for practice-site- and ACO-level covariates.

	Unadjusted	Practice Site Level	Practice Site and ACO Levels
Measures of clustering and variation			
Accountable care organization (ACO)-level variance	0.37	0.40	0.04
Intraclass correlation coefficients (ICCs)	0.10	0.11	0.01
Median odds ratios (95% credible intervals)	1.78 (1.57–3.69)	1.83 (1.56–5.00)	1.20 (1.00–4.21)
Practice-site-level measures, odds ratios			
Group practice vs. health Center		2.23 (0.84–6.48)	1.52 (0.51–4.57)
Experience with ACO contracts vs. no experience		1.56 (0.79–3.08)	1.96 (0.95–4.03)
Less than half of patients part of risk contracts vs. half or more		1.16 (0.61–2.20)	1.20 (0.64–2.26)
No experience with the same clinical partners vs. some experience		1.24 (0.66–2.32)	1.24 (0.67–2.29)
Both pediatric and adult patients at the site vs. adults only		5.11 (2.13–12.3)	5.24 (2.17–12.7)
Pediatric-only vs. adult-only		1.68 (0.61–4.65)	1.67 (0.60–4.64)
Fewer than 500 Medicaid patients vs. more than 500		1.77 (0.90–3.47)	1.67 (0.86–3.24)
ACO-level measures, odds ratios			
Teaching-hospital- vs. physician-organization-anchored			1.72 (0.71–4.16)
Community-hospital- vs. physician-organization-anchored			3.42 (1.07–11.0)
Medium vs. small organization			2.32 (0.88–6.11)
Large vs. small organization			1.07 (0.30–3.81)

Notes: ACO = accountable care organization. Cumulative odds ratios, presented for practice-site- and ACO-level measures, convey the magnitude of increased odds of reporting that delivering high-quality care became easier. The three models allow for comparisons across measures of clustering after accounting for characteristics of both the practice sites and the ACOs under which they operate. The odds ratios for each of those characteristics allow for comparisons to the MORs of the same model. Odds ratios with a greater magnitude (greater than 1.83 for the practice-site-level model; greater than 1.20 for the model with both levels) indicate characteristics that are better predictors of practice sites’ self-reported ability to provide high-quality care than the ACO under which they operate.

## Data Availability

Data are contained within the article.
